# Estimation of plant sampling uncertainty: an example based on chemical analysis of moss samples

**DOI:** 10.1007/s11356-016-7477-4

**Published:** 2016-08-24

**Authors:** Sabina Dołęgowska

**Affiliations:** Geochemistry and the Environment Division, Institute of Chemistry, Jan Kochanowski University, 15G Świętokrzyska St., 25-406 Kielce, Poland

**Keywords:** *Pleurozium schreberi* (Brid.) Mitt, Trace elements, Sampling uncertainty, Statistical methods

## Abstract

In order to estimate the level of uncertainty arising from sampling, 54 samples (primary and duplicate) of the moss species *Pleurozium schreberi* (Brid.) Mitt. were collected within three forested areas (Wierna Rzeka, Piaski, Posłowice Range) in the Holy Cross Mountains (south-central Poland). During the fieldwork, each primary sample composed of 8 to 10 increments (subsamples) was taken over an area of 10 m^2^ whereas duplicate samples were collected in the same way at a distance of 1–2 m. Subsequently, all samples were triple rinsed with deionized water, dried, milled, and digested (8 mL HNO_3_ (1:1) + 1 mL 30 % H_2_O_2_) in a closed microwave system Multiwave 3000. The prepared solutions were analyzed twice for Cu, Fe, Mn, and Zn using FAAS and GFAAS techniques. All datasets were checked for normality and for normally distributed elements (Cu from Piaski, Zn from Posłowice, Fe, Zn from Wierna Rzeka). The sampling uncertainty was computed with (i) classical ANOVA, (ii) classical RANOVA, (iii) modified RANOVA, and (iv) range statistics. For the remaining elements, the sampling uncertainty was calculated with traditional and/or modified RANOVA (if the amount of outliers did not exceed 10 %) or classical ANOVA after Box-Cox transformation (if the amount of outliers exceeded 10 %). The highest concentrations of all elements were found in moss samples from Piaski, whereas the sampling uncertainty calculated with different statistical methods ranged from 4.1 to 22 %.

## Introduction

Since the 1960s, monitoring studies using living organisms has been one of the most popular methods used to measure response of individual organism to pollutants and to assess the environmental quality (Čeburnis and Steinnes 2000; Gerhardt 2002; Wolterbeek 2002; Szczepaniak and Biziuk 2003; Burger 2006; Samecka-Cymerman et al. 2006; Zechmeister et al. 2006). Among the wide and spread group of organisms, some moss species, e.g., *Pleurozium schreberi*, *Hylocomium splendens*, *Hypnum cupressiforme*, and *Pseudoscleropodium purum* have successfully been used as bioindicators of trace elements (Kaasik and Liiv 2007; Batzias and Siontorou 2008; Dragović and Mihailović 2009; González-Miqueo et al. 2010; Kłos et al. 2011; Mariet et al. 2011) including rare earth elements (Chiarenzelli et al. 2001; Dołęgowska and Migaszewski 2013), organic pollutants (Chiarenzelli et al. 2001; Orliński 2002; Ares et al. 2009; Foan et al. 2010; Dołęgowska and Migaszewski 2011), and isotopes (Wadleigh 2003; Liu et al. 2008; Xiao et al. 2010; Migaszewski et al. 2010; Liu et al. 2011; Castorina and Masi 2015).

Environmental monitoring is a complex process which consists of many interdependent steps, so we must be aware about errors that can be introduced during a sequential treatment of sample. Each step from selection of sampling sites through sampling to chemical analysis and data interpretation has to be thought over, and all errors that come out at each of these stages should be identified and well recognized because they can be a source of partial uncertainty (Wolterbeek and Verburg 2002; Pasławski and Migaszewski 2006; Sakalys et al. 2009; Kłos et al. 2011, 2012).

In the environment, the concentration of a single element is determined by a multitude processes that may overlap and make the interpretation of results much harder. The most important parameter that describes the quality of measurement is the measurement uncertainty that involves sampling and chemical analysis (Ramsey and Ellison 2007). According to Ramsey (1998), the total uncertainty (expressed as a standard deviation) is a sum of geochemical and measurement uncertainty whereas the measurement uncertainty is a sum of sampling and analytical uncertainty. In this approach, the analytical uncertainty refers to within-analysis of variance while the sampling uncertainty describes within-location variance (Dołęgowska et al. 2015). Today, the assessment of analytical uncertainty is a routine step in the analytical process whereas the assessment of uncertainty in relation to sampling may be much more problematic. The lack of information about error sources induced by plant sampling has a significant effect on interpretation and comparison of analytical results. Chemical analysis of one sample or two (primary and duplicate) samples collected within one sampling site at a distance of 1 to 2 m may give various results. Differences in element concentrations within sampling site, in other words, between primary and duplicate samples may considerably affect the final result. The error related to sampling may even reach 70–80 %, so the estimation of sampling uncertainty is a crucial task (Ramsey and Ellison, 2007). The sample cannot be treated as an individual unrelated to sampling site and sampling procedure. Its chemistry depends on many individual and environmental factors which are beyond our control, but we can decide about type of sampling procedure and its consistency. According to Pasławski and Migaszewski (2006), the sampling uncertainty among all components has the greatest contribution to the measurement uncertainty and it should not exceed 30 % whereas in practice the ratio *s* ^2^_meas_/*s* ^2^_total_ should be lower than 20 % (Zhou et al. 2014). Understanding the relationship between element concentration and (i) environmental, physiological, and genetic factors, or (ii) sampling parameters, is a key to proper interpretation of results.

The most popular method used for calculation of sampling uncertainty is a one-way analysis of variance, which is based on the assumption that all data are normally distributed. However, many authors have indicated (Reimann and Filzmoser 2000) that this requirement is rarely fulfilled, so much more popular is a robust equivalent of classical analysis, known as the RANOVA method, which is more resistant to extreme values. Both these methods define the variance as the square of the standard deviation. However, the robust analysis of variance is down-weighting the outliers during the calculation process, so it can give more reliable results when the data show distribution that diverges from normality (Rostron and Ramsey 2012). Another, but less frequently used, is a range statistics method. It is based on the difference between the lowest and highest values. Like the one-way ANOVA, it requires a normally distributed dataset, so it can be used when the element concentration does not vary significantly within the sampling position. The one-way ANOVA and range statistics can be applied only if the preliminary assumption of normal distribution is fulfilled, whereas the RAVONA statistics is employed when the amount of outliers in a dataset does not exceed 10 % of the total results. In any other case, the data have to be transformed to achieve normality. However, the interpretation of results can be problematic despite having performed the back-transformation of obtained results (Dołęgowska et al. 2015).

The aims of the present study were to (i) highlight the differences in selected element concentrations determined in the moss species *P. schreberi* (Brid.) Mitt. collected within three forested areas and (ii) compute and compare the level of uncertainty arising from sampling using one-way ANOVA, classical and modified RANOVA, and range statistics.

## Experimental

### Study area and fieldwork

**Fig. 1:**
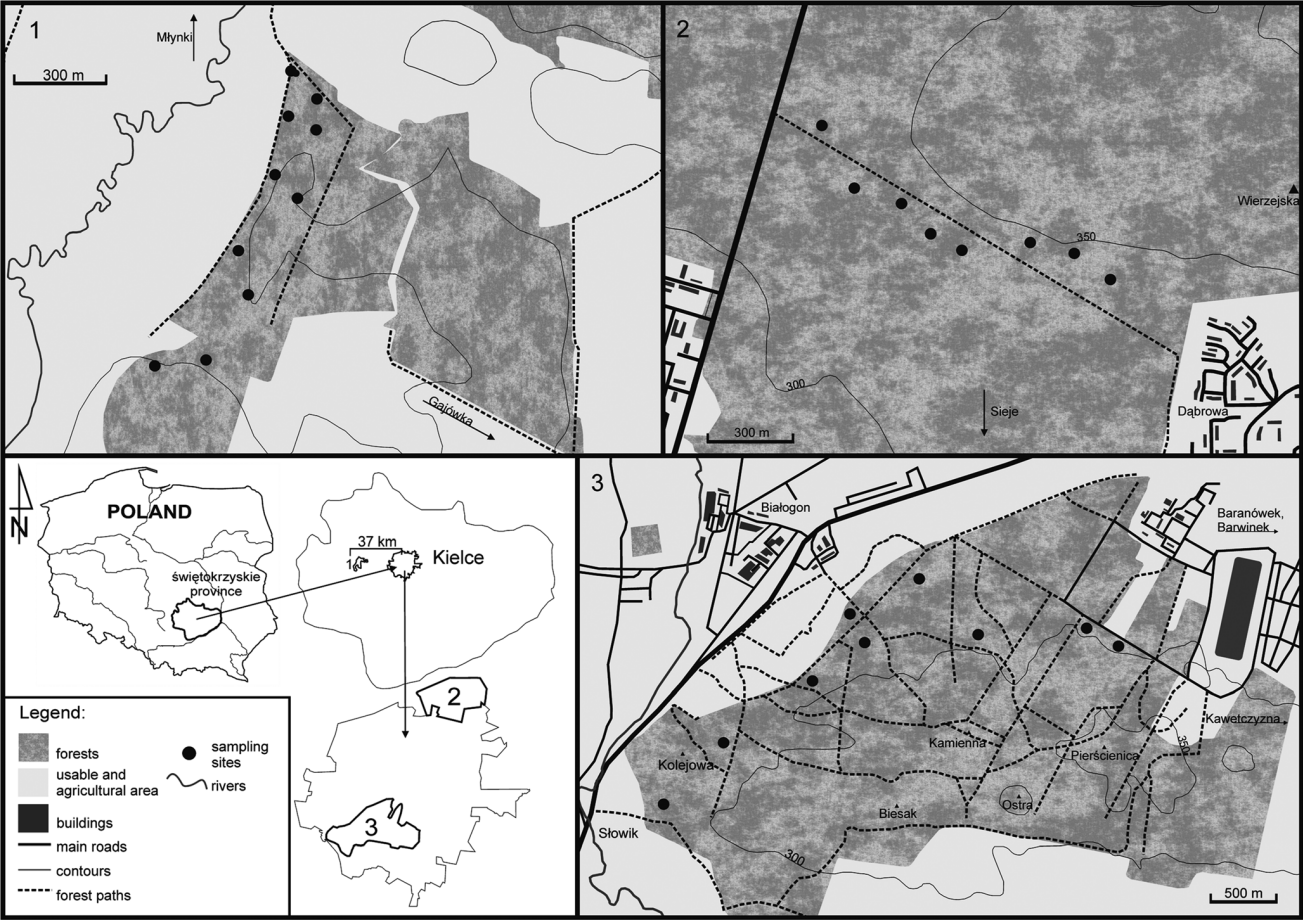
Localization of investigation sites. *1*—Wierna Rzeka. *2*—Piaski. *3*—Posłowice Range

The city of Kielce is the capital of the Świętokrzyskie province. It is located in the south-central part of Poland, in the central part of the Holy Cross Mountains (HCM). The HCM belong to a separate climatic region with average annual temperatures lower compared to surrounding lowlands. The city is situated within hills and valleys surrounded by forests; hence, this localization gives a great opportunity to conduct biomonitoring studies with naturally growing moss species. For the purpose of this study, three different wooded areas were selected (Fig. 1): (1) Wierna Rzeka located 37 km west of Kielce, selected as a pristine and a reference area (Dołęgowska et al. 2013); (2) Piaski situated in the northwesternern part of the city near the penitentiary and the local Kielce-Zagnańsk road; and (3) the Posłowice Range surrounding the southwestern part of the city, close to the Barwinek and Baranówek housing developments and the main road 762. Fieldwork was carried out in September of 2014. A total of 54 composite and duplicate samples of the moss species *P. schreberi* (Brid.) Mitt. were collected within the selected areas. Each composite sample consisted of 8 to 10 increments taken over an open space area of 10 m^2^ and mixed up to make a single sample. Duplicate samples were collected at a distance of 1 to 2 m, using the same sampling protocol (Jung and Thornton 1997). Only apical green parts of moss samples were collected, subsequently in situ cleaned from foreign organic material, placed in disposable polyethylene bags, and transported to the laboratory. To avoid influence of stemflow and throughfall, samples were collected outside of the crown projection of trees.

### Sample preparation and chemical analysis

At the laboratory, samples were stored at an ambient temperature (about 20 °C). After drying, the moss samples were triple rinsed with deionized water to remove outer contamination such as cobwebs, pollens, loosely attached mineral particles, and tiny organic material followed by drying at an ambient temperature. To avoid changes in the equilibrium of extracellular-bound cations, the time of rinsing was less than 30 s (Fernández et al. 2015).

Subsequently, samples were milled in an IKA WERKE laboratory mill to a pass <0.5-mm sieve and digested in a closed microwave system Multiwave 3000 using HNO_3_ (1:1)/H_2_O_2_ solution in the ratio of 8 mL/1 mL. The digested samples were analyzed twice for Fe, Mn, and Zn using the FAAS technique and for Cu using the GFAAS technique (THERMO SCIENTIFIC model iCE 3500Z spectrometer). Instrumental and data acquisition parameters of the digestion and AAS instrument are summarized in Table 1. As a standard reference material, tomato leaves (SRM-1573a) were used for quality control purposes. During the analysis, the recalibration process was done after a series of 10 samples analyzed. Recovery, limit of quantification (LOQ), and limit of determination (LOD) are presented in Table 1. The analytical bias was also calculated to confirm that it was not a significant part of uncertainty.

**Table 1:** Parameters of digestion process and AAS instrument

Digestion parameters		Technique	Fe	Mn	Zn	Cu
			Flame			Cuvette
Power	1000 W	Type of work	Absorption			
Time	65 min	Wave length	248.3 nm	279.5 nm	213.9 nm	324.8 nm
Time of growth	15	Type of flame	C_2_H_2_			–
Time of real digestion	30	Type of purge gas	–			Ar
Time of cooling	20	Gas flow	0.9 L min^−1^	1.0 L min^−1^	0.8 L min^−1^	0.2 L min^−1^
Temperature	220 °C	Background correction	D2			Zeeman
Pressure	6 MPa	Gap	0.2 nm			0.5 nm
*p* growth rate	0.03 MPa s^−1^	Replicates	3			
		Matrix modifier	–	–	–	1 % NH_4_NO_3_
Reagents	HNO_3_ (1:1) 8 mL	Concentration range of standard solutions	0.5–6.0 mg L^−1^	0.1–2.0 mg L^−1^	0.05–0.50 mg L^−1^	1–10 μg L^−1^
	H_2_O_2_ 1 mL					
				0.5–6.0 mg L^−1^ for Piaski		
		LOD	0.05 mg L^−1^	0.01 mg L^−1^	0.004 mg L^−1^	0.06 μg L^−1^
		LOQ	0.15 mg L^−1^	0.03 mg L^−1^	0.01 mg L^−1^	0.18 μg L^−1^
		Analytical bias	−0.02 mg L^−1^	−0.02 mg L^−1^	−0.002 mg L^−1^	−0.02 μg L^−1^
		Recovery (%)	97 %	98 %	99 %	98 %

### Statistical analysis

**Fig. 2:**
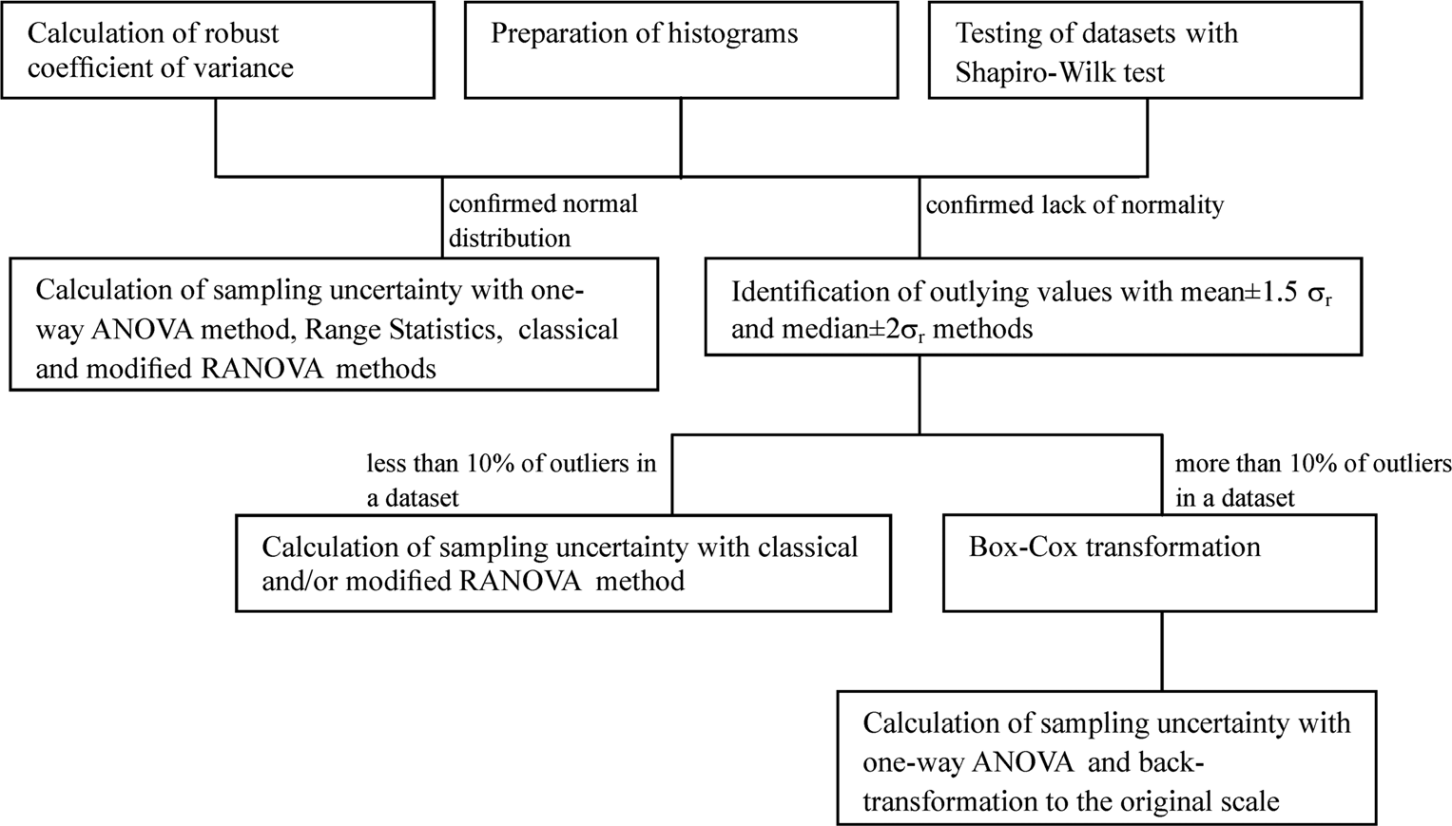
Scheme of statistical proceeding

The statistical proceeding included several steps that allowed for estimation of sampling uncertainty with all precautions (Fig. 2). In the RANOVA method, the outlying values are defined as values exceeding the relation mean *± c · σ*_*r*_ (where *σ*_*r*_ is a robust standard deviation) and during the calculation process, these are replaced by it, whereas the presence of outlying values in a dataset strongly affects the average value and change the measurement precision. To avoid the direct relation to the arithmetic mean, the outlying values were also identified by the median *± 2 · σ*_*r*_ and during the calculation process, these were replaced by it. Subsequently, the median (if it changed) and the robust standard deviation were recalculated. After each operation, the histograms were made and datasets were tested for normality. The statistical operation was repeated as *p* value (calculated with Shapiro-Wilk test) was constant, or when normality was achieved.

## Results and discussion

Table 2 shows minimum, maximum, mean, and standard deviation values computed for the elements determined in *P. schreberi* moss samples derived from three selected areas. Some statistical parameters such as *p* values (95 %) computed by Shapiro-Wilk test, skewness, and robust coefficient of variance (CV_*r*_) were also calculated. The sampling uncertainty expressed as a relative standard deviation (*s*_*r*samp_ (%)) calculated for Cu, Fe, Mn and Zn using various statistical methods is presented in Table 3.

**Table 2:** Summary statistics and statistical parameters calculated for elements from Wierna Rzeka, Piaski, and Posłowice areas

Element	Max	Min	Mean	*σ*	Skewness	*p*	CV_*r*_	Max	Min	Mean	*σ*	Skewness	*p*	CV_*r*_	Max	Min	Mean	*σ*	Skewness	*p*	CV_*r*_
	mg kg^−1^						%	mg kg^−1^						%	mg kg^−1^						%
	Wierna Rzeka							Piaski							Posłowice Range						
Cu	6.89	4.25	5.69	0.81	−0.20	0.04	24	14.35	5.98	9.80	2.05	0.14	0.56	24	12.00	5.67	7.47	1.49	1.43	0.0005	26
Fe	516.9	163.2	304.8	91.7	0.55	0.12	42	1069.7	300.3	594.5	176.7	0.79	0.01	33	521.0	199.3	341.3	101.6	0.44	0.004	59
Mn	170.8	56.3	98.9	33.1	0.68	0.005	59	1040.5	380.6	660.0	164.4	0.79	0.03	27	135.8	30.1	83.2	35.9	0.04	0.008	83
Zn	26.52	42.54	34.45	4.15	0.02	0.35	12	68.34	37.05	48.65	9.16	0.61	0.03	32	48.69	32.58	40.29	5.04	0.02	0.05	26

*σ*—standard deviation calculated for each element

**Table 3:** Sampling uncertainty (expressed as relative standard deviation—*s*_*r*samp_ (%)) calculated for elements from Wierna Rzeka, Piaski, and Posłowice using different statistical methods

Element	ANOVA	Range statistic	RANOVA	Modified RANOVA	ANOVA after Box-Cox transformation	ANOVA	Range statistic	RANOVA	Modified RANOVA	ANOVA after Box-Cox transformation	ANOVA	Range statistic	RANOVA	Modified RANOVA	ANOVA after Box-Cox transformation
	*s*_*r*_ (%)	*s*_*r*_ (%)	*s*_*r*_ (%)	*s*_*r*_ (%)	*s*_*r*_ (%)	*s*_*r*_ (%)	*s*_*r*_ (%)	*s*_*r*_ (%)	*s*_*r*_ (%)	*s*_*r*_ (%)	*s*_*r*_ (%)	*s*_*r*_ (%)	*s*_*r*_ (%)	*s*_*r*_ (%)	*s*_*r*_ (%)
	Wierna Rzeka					Piaski					Posłowice Range				
Cu	–	–	–	12	–	15	14	15	12	–	–	–	–	11	–
Fe	22	22	21	18	–	–	–	–	17	–	–	–		–	5.5
Mn	–	–	–	–	7.7	–	–	–	–	4.1	–	–		–	9.7
Zn	7.8	8.7	8.7	7.1	–	–	–	–	4.3	–	12	13	13	13	–

*s*_*r*_ (%)—uncertainty expressed as relative standard deviation

### Differences in element concentrations between study areas

All the determined elements are life-essential for plants (Grodzińska et al. 2003), for example, Zn regulates biomass growth whereas Mn plays a role in oxidation/reduction processes and electron transport in photosynthesis. This also activates many enzymes and imposes Fe deficiency. Fe is a component of enzymes and proteins; it is responsible for respiration and photosynthesis as a factor regulating a synthesis of chlorophyll. In plants, copper regulates many physiological processes and is a factor of metalloproteins (Miller et al. 1995; Hafeez et al. 2013; Sadeghzadeh 2013). According to moss bioindicative properties, the higher content of this element in the air is the higher its concentration in moss tissues. In this study, the moss samples from Piaski were enriched in all the elements examined. The Mn content was even three times higher than in samples from Posłowice and Wierna Rzeka (Table 2). The concentration of Mn in lichens *Hypogymnia physodes* and 1- and 2-year-old pine needles collected at nearby Wierzejska Mt. (375 m a.s.l.) in 1994 and 1995 were as follows: lichens (sampled from different trees)—53 and 144 mg kg^−1^ and needles—depending on needle age—320 and 455 mg kg^−1^ (Migaszewski and coauthors, unpubl. data). The chemical analysis of *P. schreberi* samples performed in 2009 also gave much lower levels, 219 and 234 mg kg^−1^, respectively (Dołęgowska et al. 2013). These differences may arise from a negative correlation between element concentrations and altitude. However, the results derived from this study are not unequivocal (Sucharová and Suchara 2004; Coşkun et al. 2005; Gerdol and Bragazza 2006). The mean concentration of Mn in the moss species *H. splendens* and *P. schreberi* from the Holy Cross Mountains reported by Gałuszka (2007) was 364 mg kg^−1^. The average content of Mn (660 mg/kg) in moss samples from Piaski is much higher than that noted in mosses from other regions of Poland: Silesia-Kraków—145 mg kg^−1^, Legnica-Głogów—278 mg kg^−1^ (Grodzińska et al. 2003), Stalowa Wola—∼250 mg kg^−1^ (Samecka-Cymerman et al. 2006), or neighboring countries: Russia—300 mg kg^−1^ (Ermakova et al. 2004), Czech Republic—416 mg kg^−1^ (Sakalys et al. 2009), Germany—331 mg kg^−1^ (Siewers et al. 2000), Lithuania—273 mg kg^−1^ (Čeburnis and Steinnes 2000), and also in Macedonia—186 mg kg^−1^ (Barandovski et al. 2008), Bułgaria—251 mg kg^−1^ (Stamenov et al. 2002), and Spain—285 mg kg^−1^ (*S. purum*) and 210 mg kg^−1^ (*H. cupressiforme*) (Fernández and Carballeira 2002). The similar average values were noted only in mosses from Norway—542 mg kg^−1^ (Reimann et al. 2006) and France—712 mg kg^−1^ (Leblond et al. 2004). The concentrations of trace elements in the mosses may vary with time because of a selective loss of elements, but Mn enrichment results predominantly from increased Mn cycling in forest ecosystems. Along with Zn, Mn occurs in throughfall in a dissolved fraction, so the enrichment may also come from the recretion of the canopy (Gandois et al. 2010a). What is more, Mn shows a strong affinity for organic matter, so it can be retained in moss tissues in higher concentrations.

The comparison of Fe, Mn, Cu, and Zn concentrations in various tissues of *Pinus sylvestris* shows that Mn and Zn accumulate mainly in 2- and 3-year-old needles whereas Fe in pine bark (Pasławski and Migaszewski 2006). Dragović and Mihailović (2009) reported that Mn and Zn accumulate in mosses by leaching from higher plants (and additionally from throughfall); this process is another significant source of these elements. In practice, to avoid the influence of throughfall and leaching from higher plants on moss chemistry, samples should be collected, if possible, in open space areas.

The enrichment in Mn and Zn observed in the mosses from Piaski is also induced by different soil conditions (acidity). The average ${\mathrm{pH}}_{{\mathrm{CaCl}}_2}$ of soil samples from Piaski is 3.1, whereas of soils from Posłowice and Wierna Rzeka 4.1 and 4.2, respectively. The lower pH makes these elements more mobile and bioavailable (Gandois et al. 2010b). Mosses do not have a root system; therefore, these elements are transported with airborne soil particles and adsorbed onto mosses; this is the reason why they can be much easier washed into moss tissues. Another aspect that should be taken under consideration is a probable correlation between moss occurrence and bioaccumulation process. Pesch and Schröder (2006) noticed that rare moss occurrence is accompanied by high bioaccumulation process and vice versa. Of the three study areas (Piaski, Posłowice, and Wierna Rzeka), the Piaski area is characterized by the lowest moss coverage and the highest environmental degradation. The soil profile is shallow and underdeveloped. The poorer soil coverage increases the amount of particles that may be freely transported and deposited onto moss tissues. The same relationship between the stable sulfur isotope signature and moss coverage was also reported by Migaszewski et al. (2010). Where the coverage of the moss carpet was extensive, the δ^34^S values were high, and vice versa. Sites with thick tangled moss mats showed the lowest δ^34^S and a lack of distinct isotope diversity between the moss species *H. splendens* and *P. schreberi*, and vice versa. The diverse moss density may also result from various microclimatic and edaphic conditions (Holy et al. 2009).

Pasławski and Migaszewski (2006) found the highest concentrations of Fe in the pine bark and epiphytic lichens growing on these trees and the lowest in the pine needles and roots. The significant disproportionation between the element content and various tissues was not observed for Cu. However, the highest concentrations of this metal were observed in the 1-year pine needles. Both Fe and Cu represent the soil contribution and are accumulated by higher plants from dry deposition. Except for throughfall, dry deposition was also reported by Dragović and Mihailović (2009) as a main source of Fe and Cu in mosses. The content of Fe in the moss samples from the Posłowice Range and Wierna Rzeka is similar to that reported for national parks of Poland (Grodzińska et al. 1999) and other Central-European countries (Harmens et al. 2010). By contrast to Posłowice and Wierna Rzeka, the samples from Piaski were enriched in this element. The higher concentrations of Fe were close to those found in industrial regions of Poland (1226 mg kg^−1^) and some European countries (Belgium, Slovenia, Italy, and France). The levels of Cu (Table 2) were in turn typical of those noted in mosses from the other European regions (Grodzińska et al. 2003; Harmens et al. 2010).

The element concentrations in the mosses from Posłowice and Wierna Rzeka fall into the following concentration sequence: Fe> > Mn > Zn > Cu whereas in the moss samples from Piaski, this element trend is different: Mn > Fe > Zn > Cu. The same Posłowice and Wierna Rzeka sequence revealed the moss samples collected from other parts of the HCM (Gałuszka 2007; Dołęgowska et al. 2013), and throughout the world, e.g., Serbia (Dragović and Mihailović 2009), Romania (State et al. 2012), France (Gandois et al. 2014), Finland (Salo et al. 2012), and Argentina (Wannaz et al. 2012).

There is no correlation between the coefficient of variation and the concentration level. The robust coefficient of variation (CV_*r*_) calculated for all the determined elements varied from 12 to 83 %. The highest variability was noted for Mn and Fe from Posłowice (83 %, 59 %) and Mn from Wierna Rzeka (59 %). The results below 30 % were obtained for Cu and Zn from Posłowice (26 %) and Wierna Rzeka (24 %, 12 %) and for Cu and Mn from Piaski (24 %, 27 %). The CV_*r*_ values for the remaining elements were in the range of 30 to 42 %. The highest coefficient of variation computed for the moss samples from the Kielce region was also noted for Mn—61 % whereas for Cu, Fe, and Zn averaged around 20 % (Dołęgowska et al. 2013). The highest variability of Mn in mosses was also observed by Gałuszka (2007) (about 100 %) and by Reimann et al. (2006) (64 %).

### Sampling uncertainty

Sampling is one of the most important contributions to the uncertainty (Ramsey and Thompson 2007; Dołęgowska and Migaszewski 2015). It is a part of measurement uncertainty and can be calculated as a difference between measurement and analysis sum of square (Ramsey 1998). To prove that chemical analysis was not a significant source of uncertainty, the analytical bias was also calculated (Table 1). Moreover, to reduce the analytical bias, the equipment was checked and calibrated properly for the range of expected values (Table 1). According to the literature, the analytical method is fit for purpose when the *s* ^2^_anal_ is less than 20 % of *s* ^2^_meas_ (Ramsey et al. 1992). For all the elements examined, this relation was met, so the analytical procedure was fit for purpose and was not a significant source of uncertainty.

As mentioned before, the classical analysis of variance can be applied when the datasets fulfill the assumption of normality; otherwise, robust models need to be used or data need to be transformed. Rejection of outlying values is not recommended because in environmental studies, they may carry a crucial information about the study area and potential “hot spots” (Gałuszka et al. 2015). In this study, computed coefficients of skewness and shapes of histograms confirmed asymmetrical distribution of the majority of analyzed elements (Table 2). The normal distribution was only approached for Cu—Piaski, Zn—Posłowice, and Fe and Zn—Wierna Rzeka. Therefore, only for these elements, the sampling uncertainty was computed with the one-way ANOVA, range statistics, traditional, and modified RANOVA methods. The lowest *s*_*r*samp_ values (except for Zn-Posłowice) were obtained with modified RANOVA method but the differences between all values were in the range of 1 to 4 % (Table 3). The comparison of classical ANOVA and RANOVA showed that higher values were typically given by RAVONA whereas the range statistics method gives intermediate values (except for Cu—Piaski).

The same relationship was noted by the other authors (Gron et al. 2007; Zhou et al. 2014), but as shown by Dołęgowska et al. (2015), it is not always preserved. However, it should be stressed that most studies have encompassed soil or food samples. The uncertainty arising from plant sampling was computed by Smagunova et al. (2004) and Lyn et al (2007). Pasławski and Migaszewski (2006) reported that the uncertainty of sampling should not exceed 20 %, but usually is in the range of 10 to 30 %, whereas Ramsey et al. (1992) assumed that if the whole procedure is to be fit for purpose, the combined sampling and analytical variances (measurement variances) for the data must comprise less than 20 % of the total variance. In this study, the sampling uncertainty higher than 20 % was obtained for Fe from Wierna Rzeka. These results are consistent with the high robust coefficient of variance (42 %), so it can be expected that the high sampling uncertainty arises from the large variability of this element in the environment and not from incorrect sampling. The same relationship was found for Zn from Wierna Rzeka where the lowest sampling uncertainty (7.1 %) corresponded with the lowest coefficient of variation (12 %). As for Zn from Posłowice and Cu from Piaski, the sampling uncertainty was in the range of 12–13 and 12–15 %, respectively. For these two elements, the robust coefficient of variation was 26 and 24 %.

With respect to Fe and Zn from Piaski and Cu from Posłowice and Wierna Rzeka, the data distribution diverged from normal. The amount of outlying values identified with mean *± 1.5σ*_*r*_ (where *σ*_*r*_ is a robust standard deviation) exceeded 10 %, whereas that identified with median *± 2σ*_*r*_ was below 10 %, so to avoid data transformation, the sampling uncertainty was computed with the modified RANOVA method (using median *±* 2*σ*_*r*_ instead of mean *±* 1.5*σ*_*r*_) (Table 3). The *s*_*r*samp_ values computed with different methods are presented in Table 3. The highest uncertainty was noted for Fe and the lowest for Zn. It is interesting to note that Zn and Fe have a higher coefficient of variation compared to Cu. The dispersion of results lead to a greater difference between mean and median values; hence, the higher differences are found between the *s*_*r*samp_ values. The same results were obtained by Smagunova et al. (2004). These authors found the highest sampling uncertainty for Fe (20–25 %) and Mn (9.9–33 %), whereas the lowest for Zn (about 7.7 %). The analysis of duplicate samples of spruce needles gave the sampling uncertainty of 13 % for Mn and 6 % for Zn (Čeburnis and Steinnes 2000). However, the highest uncertainty exceeding 25 % was obtained for As (29 %) and V (21 %).

In general, the robust statistical techniques are more adequate for analysis of environmental results. These datasets are scarcely devoid of extreme values and their presence arises mainly from natural geochemical diversity. The use of median *± c · σ*_*r*_ instead of mean *± c · σ*_*r*_ during the calculation process allows us to avoid the direct relationship with the arithmetic mean, which is strongly dependent on outliers.

In case of Fe and Mn from Posłowice and Mn from Piaski and Wierna Rzeka, the number of outliers exceeded 10 % of the total results (identified with mean *±* 1.5*σ*_*r*_ and median *±* 2*σ*_*r*_); therefore, the datasets were transformed using the Box-Cox function. To compare the sampling uncertainty (computed with the transformed data) with the values obtained for the raw dataset, the back-transformation process was conducted. The sampling uncertainty computed for the transformed data with the one-way ANOVA method was 5.5 % for Fe and ranged from 4.1 to 9.7 % for Mn (Table 3). The *s*_*r*samp_ values were much lower compared to those obtained for the raw data. However, the relationship between the lower coefficient of variance and the lower sampling uncertainty was kept. As shown by Dołęgowska et al. (2015), the transformation reduces non-normality, but gives a completely different dataset. The use of back-transformation allows comparison of results with raw data. Nonetheless, this introduces another operation on the data. It is noteworthy that the use of log_10_x transformation gave the *s*_*r*samp_ at the same level. The Box-Cox function was much suitable and allowed for achieving normality for all the datasets tested. The log_10_x transformation failed to restore normality for Fe from Piaski and Cu from Wierna Rzeka.

## Conclusions

The results derived from this study enable drawing the following conclusions:

1. The moss samples from Piaski were enriched in all the elements examined and the Mn concentrations were even three times higher than those in samples from Posłowice and Wierna Rzeka. This enrichment may be linked to a lower soil pH and sparse moss occurrence.
2. The highest coefficients of variance were noted for Mn and Fe from Posłowice and Wierna Rzeka.
3. The following relationship was observed: the higher coefficient of variance, the higher sampling uncertainty. This relation is logical, although it was not observed for all the elements examined.
4. The comparison of classical ANOVA, RANOVA, and range statistics showed that the *s*_*r*samp_ values computed with the range statistics method (except for Cu from Piaski) are between those obtained with the other two statistical methods. This method could be more suitable for normally distributed datasets.
5. The comparison of classical and modified RANOVA methods showed that using median *± c · σ*_*r*_ instead of mean *± c · σ*_*r*_ during the calculation process leads to lower *s*_*r*samp_ values. This also enables us to avoid a direct relationship with the arithmetic mean, which is strongly dependent on outliers. This model could be more appropriate for datasets with distribution diverging from normal.
6. The use of Box-Cox transformation helps achieve normality and enables us to calculate uncertainty with the one-way ANOVA method. The back-transformation to the original scale gives the possibility to compare our results with the raw data. This transformation can be used when the others, such as log_10_ transformation, do not lead to achieve normality.
7. The *s*_*r*samp_ values computed for the transformed data are much lower compared with the raw data (despite the back-transformation process), and their interpretation may be more dubious.
